# Combined analysis of chromatin accessibility and gene expression profiles provide insight into Fucoxanthin biosynthesis in *Isochrysis galbana* under green light

**DOI:** 10.3389/fmicb.2023.1101681

**Published:** 2023-02-10

**Authors:** Duo Chen, Huan Li, Jing Chen, Yuying Han, Xuehai Zheng, Yixin Xiao, Xupeng Chen, Tao Chen, Jiannan Chen, Youqiang Chen, Ting Xue

**Affiliations:** The Public Service Platform for Industrialization Development Technology of Marine Biological Medicine and Products of the State Oceanic Administration, Center of Engineering Technology Research for Microalga Germplasm Improvement of Fujian, Fujian Key Laboratory of Special Marine Bioresource Sustainable Utilization, Key Laboratory of Developmental and Neural Biology, Southern Institute of Oceanography, College of Life Sciences, Fujian Normal University, Fuzhou, China

**Keywords:** *Isochrysis galbana*, fucoxanthin, ATAC-seq, RNA-seq, transcription factor

## Abstract

*Isochrysis galbana*, as a potential accumulator of fucoxanthin, has become a valuable material to develop functional foods for humans. Our previous research revealed that green light effectively promotes the accumulation of fucoxanthin in *I. galbana*, but there is little research on chromatin accessibility in the process of transcriptional regulation. This study was conducted to reveal the mechanism of fucoxanthin biosynthesis in *I. galbana* under green light by analyzing promoter accessibility and gene expression profiles. Differentially accessible chromatin regions (DARs)-associated genes were enriched in carotenoid biosynthesis and photosynthesis-antenna protein formation, including *IgLHCA1*, *IgLHCA4*, *IgPDS*, *IgZ-ISO*, *IglcyB*, *IgZEP*, and *IgVDE*. The motifs for the MYB family were also identified as candidates controlling metabolic regulation responses to green light culture of *I. galbana*, including *IgMYB1*, *IgMYB2*, *IgMYB33*, *IgMYB42*, *IgMYB98*, *IgMYB118*, and *IgMYB119*. The results of differential expression analysis and WGCNA showed that several genes or transcription factors (TFs) related to carotenoid metabolism and photosynthesis exhibited a higher expression level and were significantly upregulated in A-G5d compared with A-0d and A-W5d, including *IgMYB98*, *IgLHCA1*, *IgLHCX2*, *IgLHCB4*, and *IgLHCB5*. This suggests that upregulation of these genes by green light may be the key factor leading to fucoxanthin accumulation by regulating the photosynthesis-antenna protein pathway. An integrated analysis of ATAC-seq and RNA-seq showed that 3 (*IgphoA*, *IgPKN1*, *IgOTC*) of 34 DARs-associated genes displayed obvious changes in their chromatin regions in ATAC-seq data, suggesting that these genes specific for green light may play a key role in fucoxanthin biosynthesis in *I. galbana* through a complex regulatory network of multiple metabolic pathways interacting with each other. These findings will facilitate in-depth understanding the molecular regulation mechanisms of fucoxanthin in *I. galbana* and its role in response to green light regulation, providing technical support for the construction of high fucoxanthin content strains.

## Introduction

Aquatic algae have acquired a range of adaptive mechanisms to cope with the harsh features of aquatic environments, such as poor light quality, low temperature, high salt, and low nutrition. In the long history of evolution, algae have lived in environments ranging from shallow to deep seas, where light is the most important environmental factor for the growth of microalgae. The marine microalgae *Isochrysis galbana* is a group of oxygenic photosynthetic organisms that possess fucoxanthin Chl a/c-binding proteins as light-harvesting antennae, which has exceptional blue-green light harvesting and photoprotection capabilities (Pi et al., 2019; Wang et al., 2020). *Isochrysis galbana*, as a potential accumulator of fucoxanthin, is recognized for being a rich source of fucoxanthin (more than 10% of dry weight biomass) and lipids (7.0–20.0% dry weight biomass), and has become an valuable material to develop functional foods for humans due to its small size, fast growth rate, high fucoxanthin content, and large-scale artificial cultivation (Kim, 2012; Zhu et al., 2018). Stress conditions could induced microalgae to synthesize carotenoids. Weighted gene co-expression network analysis (WGCNA) was used to investigate the underlying molecular mechanisms of *Dunaliella salina* response to salinity stress, identifying several salinity specific hub genes (Panahi and Hejazi, 2021). In order to overcome the low biomass of *Auxenochlorella protothecoides* and increase the productivity of secondary metabolites by using heterotrophic growth model, WGCNA was used to identify that some hubs, such as serine hydroxymethyltransferase (*SHMT1*), was the best candidate genes for the development of metabolites accumulating strains in microalgae (Panahi et al., 2020). Additionally, this marine microalga is much easier to process compared with other kind of algae because of its lack of a cell wall and is considered an ideal bait for the development of aquatic animals (Chen et al., 2017). Studies have shown that fucoxanthin has remarkable biological characteristics, including antioxidant, antitumor, antibacterial, antiviral, antiobesity, neuroprotective, and other pharmacological effects, which are in increasing demand in the biopharmaceutical and cosmetic fields (Maeda et al., 2015; Kim et al., 2018).

In our previous study, we generated a high quality genome assembly of *I. galbana* with a total size of ~92.73 Mb and identified several functional genes related to the fucoxanthin biosynthesis pathway, including phytoene synthase (*IgPSY*), phytoene desaturase (*IgPDS*), ζ-carotene desaturase (*Ig*ZDS), carotenoid isomerase (*Ig*CRTISO), zeaxanthin epoxidase (*IgZEP*), violaxanthin de-epoxidase (*IgVDE*), lycopene β-cyclase (*IglcyB*), 9-cis-beta-carotene 9′,10′-cleaving dioxygenase 7 (*IgCCD7*), and all-trans-10′-apo-beta-carotenal 13,14-cleaving dioxygenase (*IgCCD8*) (Chen et al., 2022). Previous studies have reported that green light has a significant effect on the metabolism of fucoxanthin in *I. galbana*, and several genes or TFs associated with the biosynthesis pathways of fucoxanthin were identified by integrating genome and the transcriptome analyses (Chen et al., 2022). Although these studies have preliminarily explored the transcriptional regulatory mechanisms of fucoxanthin in *I. galbana*, it is unknown whether chromatin remodeling is related to fucoxanthin biosynthesis and how it responds to green light-mediated culture.

An assay for transposase accessible chromatin sequencing (ATAC-seq) is an efficient technology used to study genome-wide open chromatin region accessibility by investigating chromatin status and identifying transcription factor binding sites. This technique is popular because of its simple operation, time-saving, strong repeatability, and low material input compared to ChIP-seq, DNAse-seq, and FAIRE-seq (Wei et al., 2018; Sen et al., 2021, 2022). Although ATAC-seq has been used successfully in *Arabidopsis thaliana*, *Vitis amurensis*, embryonic cells, and tumors, there is little research on chromatin accessibility for gene expression in algae (Corces et al., 2018; Del Priore et al., 2021; Farmer et al., 2021; Ren et al., 2021). In this study, ATAC-seq was used to identify the key DARs, motifs, and TFs that caused the fucoxanthin accumulation in *I. galbana* under green light treatment. Additionally, RNA-seq can truly reflect the gene expression profile at specific time points, providing an important reference for gene regulation mechanism of fucoxanthin in *I. galbana*. Integrating ATAC-seq and RNA-seq analyses can complement each other to analyze the regulation and expression of differentially expressed genes (DEGs) and their related chromatin open regions, further identifying the key regulatory elements responsible for fucoxanthin in *I. galbana*. These results can help to explore the molecular regulation mechanism of fucoxanthin in *I. galbana* and its role in response to green light regulation, providing technical support for the construction of high fucoxanthin content strains.

## Materials and methods

### Samples and treatment conditions

**Figure 1:**
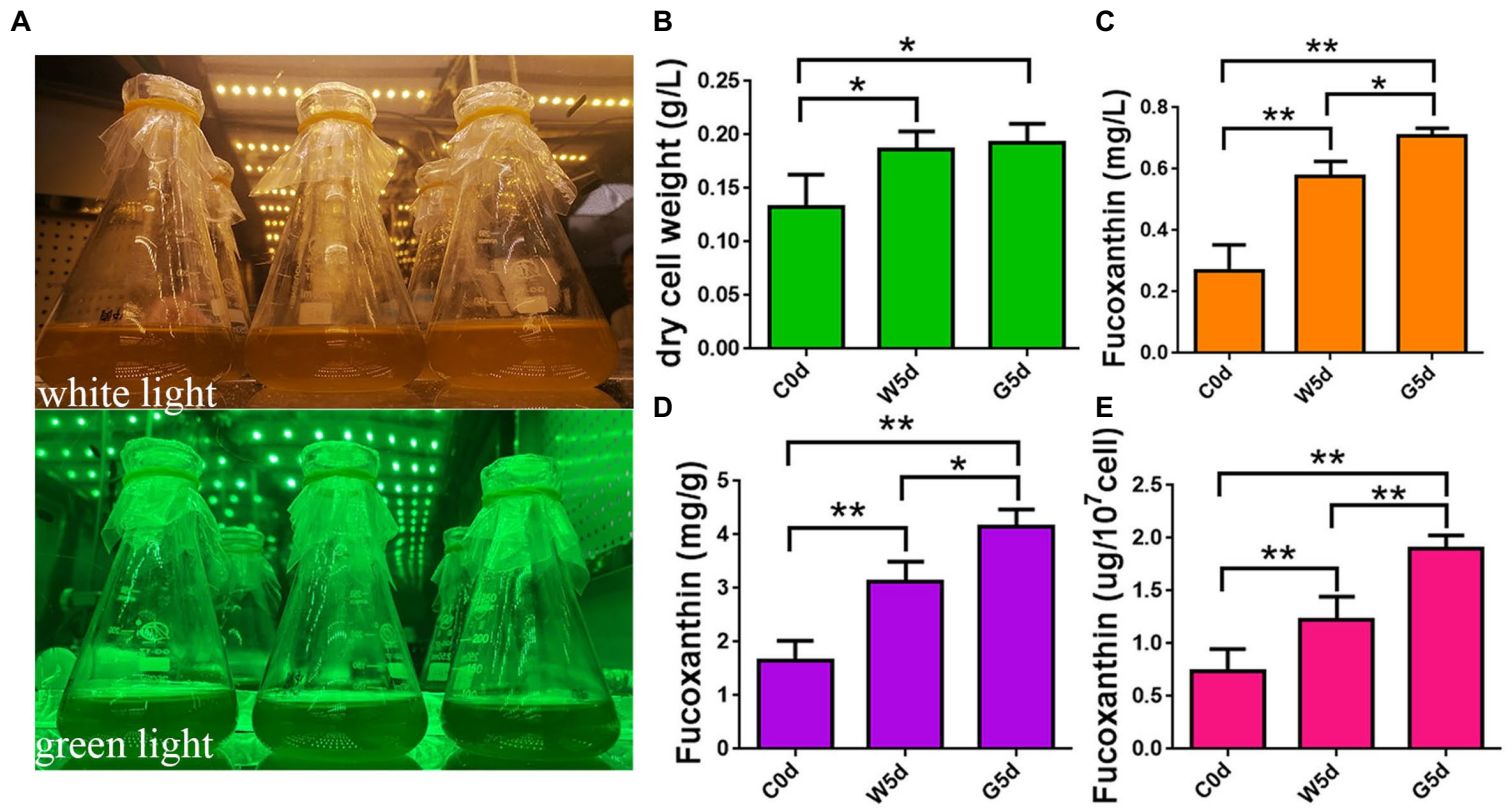
Determination of fucoxanthin content by HPLC. **(A)** Spectrum adjustable plant growth box. **(B)** Content of dry cell weight. **(C)** Content of fucoxanthin in culture system. **(D)** Content of fucoxanthin in unit dry weight. **(E)** Content of fucoxanthin in unit cell. C0d: *Isochrysis galbana* cultured under green light irradiation 100 μmol photons m^−2^ s^−1^ or white light of 100 μmol photons m^−2^ s^−1^ for 0 days. W5d: *I. galbana* cultured under white light of 100 μmol photons m^−2^ s^−1^ for 5 days. G5d: *I. galbana* cultured under green light of 100 μmol photons m^−2^ s^−1^ for 5 days. All experiments were performed in triplicate. Each value presents the mean ± SD. “I” represents error bars for the various determinations (*n* = 3). “*” and “**” indicate that significance is at 0.05 and 0.01, respectively.

*I. galbana* LG007 used in this study was separated from the near sea area of Chuanshi Island in Fujian and deposited at Fujian Normal University, China. The algae was inoculated in f/2 culture medium in conical flasks at a density of 10^6^ cells per liter. Incubation conditions were as follows: the light intensity was 100 μmol·m^−2^·s^−1^, the temperature was 23 ± 1°C, the light cycle was 24 h, and the flasks were shaken three times a day. In our previous study, the fucoxanthin yield of *I. galbana* reached its maximum at 5 days but started to decrease significantly at 7 days, indicating that Day 5 might be a key time point in fucoxanthin accumulation. When the cells reached the end of logarithm (~5 × 10^6^), the cells were divided into two groups with three biological replicates: one group remained cultured with white light of 100 μmol photons m^−2^ s^−1^ for 5 days as a control group (W5d), and the other group was cultured with green light of 100 μmol photons m^−2^ s^−1^ for 5 days as the treatment group (G5d) (Figure 1A).

### Extraction and determination of Fucoxanthin content

After vacuum freeze-drying, 1 mL of methanol was added to the freeze-dried algae with vortexing, and then left to set for at least 1 h with shaking 2–3 times a day. The supernatant was collected by centrifugation at 8,000 rpm for 15 min and filtration with a 0.25 μm filter membrane and analyzed by HPLC using a Waters 2,695 liquid chromatograph equipped with a Waters 2,998 detector (Chen et al., 2022). The operating conditions of HPLC were set as follows: column, Sunfire C18 column (4.62 mm × 50 mm, 5 μm); mobile solvent A (water), mobile solvent B (methanol), and mobile solvent C (acetonitrile); flow rate, 1 mL/min; column temperature, 40°C; injection volume, 10 μL; injection time, 16 min; and detection wavelength, 448 nm. Measurements were performed with a gradient program of mobile solvent as follows: 0 min, 15% A, 30% B, 55% C; 0-10 min, from 15 to 0% A, from 30 to 15% B, from 55 to 85% C; 10–11 min, from 15 to 10% B, from 85 to 90% C; 11–15 min, 10% B, 90% C; 15–16 min, from 0 to 15% A, from 10 to 30% B, from 90 to 55% C; 16–20 min, 15% A, 30% B, 55% C. Stock solution of fucoxanthin was dissolved with methanol solution, and gradient diluted as follows: 5, 10, 20, 40, 60, 80, and 100 μg/mL. Fucoxanthin content was determined according to the linear relationship between the peak areas (Y) and fucoxanthin content (X) of a standard curve (*R*^2^ = 0.998, *Y* = 6.88 × 10^4^X + 3.21 × 10^4^).

### ATAC sequencing and analysis

Nine samples were used for ATAC sequencing, namely A-0d-1, A-0d-2, and A-0d-3 (samples for initial culture at Day 0 under white or green light), A-W5d-1, A-W5d-2, and A-W5d-3 (samples cultured for 5 days under white light), and A-G5d-1, A-G5d-2, and A-G5d-3 (samples cultured for 5 days under green light). Cells (~5 × 10^4^) were collected by centrifugation at 7,200 rpm for 5 min and then washed two times with cold phosphate buffer solution (pH 7.4). The obtained cells were suspended with cold lysis buffer and centrifuged again with the same parameters to remove the supernatant. The isolated nuclei were resuspended with the Tn5 transposase for transposing reaction system and incubated at 37°C for 30 min, then immediately purified using a Qiagen MiniElute Kit (Qiagen, CA, United States). The PCR amplification reaction of the purified DNA was carried out as previously reported and sequenced on the Novaseq 6,000 platform with PE150. Clean reads were obtained from raw reads of Illumina by Cutadapt (version 1.8.3) with default parameters (Martin, 2011). The quality-filtered data were mapped onto the *I. galbana* LG007 genome using Bowtie2 (version 2.2.4) (Langmead and Salzberg, 2012). Density distribution of sequencing reads within the 3 kb interval up- and downstream of the TSS for each gene was performed by using DeepTools (version 2.07) (Ramírez et al., 2016). Peak calling was carried out using MACS2 (version 2.1.1) with a threshold of FDR < 0.05, then functionally annotated for genome-wide peaks by using ChIPseeker (version 1.2.6) (Zhang et al., 2008; Yu et al., 2015). MEME-ChIP (version 4.11.2) was used to identify and annotate the Motif (Machanick and Bailey, 2011). DARs were identified using DiffBind (version 2.2.11) with a threshold of fold change > = 1.2 and FDR < 0.05 (Stark and Brown, 2011). Associated genes in the DARs were compared with NR, Swiss-prot, GO, KEGG, COG, KOG, eggNOG, and Pfam databases to obtain the annotation information (Ashburner et al., 2000; Tatusov et al., 2000; Apweiler et al., 2004; Kanehisa et al., 2004; Deng et al., 2006; Finn et al., 2014; Huerta-Cepas et al., 2016). GO and KEGG enrichment analyses of DARs-associated genes in the promoter region were performed using the R package clusterProfiler (version 4.2) (Yu et al., 2012). We finally generated a total of ~1.16 Gb clean reads with Q30 ≥ 96.71%; over 86.19% of total reads were mapped to the *I. galbana* LG007 genome, indicating that the quality of data generated by sequencing is good (Supplementary Tables S1, S2).

### mRNA sequencing and analysis

Nine samples were used for mRNA sequencing, namely R-0d-1, R-0d-2, and R-0d-3 (samples for initial culture at Day 0 under white or green light), R-W5d-1, R-W5d-2, and R-W5d-3 (samples cultured for 5 days under white light), and R-G5d-1, R-G5d-2, and R-G5d-3 (samples cultured for 5 days under green light). Total RNA was extracted with TRIzol reagent (Invitrogen Life Technologies, CA, United States). A NanoPhotometer (IMPLEN, CA, United States) and a 2,100 Bioanalyzer (Agilent Technologies, United States) were used to check the purity and quality of RNA. RNA libraries were prepared as previously described and sequenced on the Novaseq 6,000 platform with PE150. Clean reads were obtained from raw reads of Illumina by Trimmomatic (version 0.36), and then mapped to the genome using HISAT2 (version 2.2.1) (Anthony et al., 2014; Pertea et al., 2016). We finally generated a total of ~116 Gb data with Q30 ≥ 91.42%, and the mapping ratio of each sample to the genome assembly ranged from 92.60 to 93.83% (Supplementary Tables S3, S4). Differentially expressed genes (DEGs) were identified by using EdgeR (version 4.2) with the criteria of |log2 Fold change| ≥1.5 and value of *p* <0.05 (Nikolayeva and Robinson, 2014). Enrichment analysis of GO and KEGG was carried out by using topGO R package (version 3.8) and KOBAS (version 3.0), respectively (Ashburner et al., 2000; Xie et al., 2011). We finally generated a total of ~116 Gb raw data with Q30 ≥ 91.42%, and the mapping ratio of each sample to the genome assembly ranged from 92.60 to 93.83% (Supplementary Tables S3, S4).

### Weighted gene coexpression network analysis

The WGCNA software package in R program was used to construct a coexpression network involved in fucoxanthin accumulation in *I. galbana* under different lights (Zhao et al., 2018). The weight values were calculated using a standardized gene expression matrix by pickSoftThreashold in the WGCNA package. We chose a soft power value β (β = 13) to approximate a scale-free network topology to generate a network, guided by a convenient 1-step network construction and module detection function in the R Tutorial (version 1.1). Then, the Module Eigengene (ME) was calculated, which represents the expression profile of each module. Next, based on the correlation between the ME and trait, we estimated the module-trait relationships to identify highly correlated modules. The module is considered to be associated with traits, where the moduletrait relationship value is ≥0.6 and *p* ≤ 0.05. The softConnectivity function was used to calculate the connectivity degree of genes, and the top 10 genes in the module were selected as the core genes of the module.

### Correlation analysis of mRNA and ATAC sequencing

Expression profiles of differentially peak-related genes in ATAC were combined with DEGs in RNA-seq. When multiple differential peaks were associated with the same gene, the highest peak proximal to the gene was selected. Upregulated DEGs from RNA-seq data were compared to the related genes with up-egulated peaks in ATAC. Downregulated DEGs from RNA-seq data were compared to the related genes with downregulated peaks in ATAC. Selected candidate genes were analyzed for GO and KEGG enrichment analysis by using topGO R package (version 3.8) and KOBAS (version 3.0), respectively (Ashburner et al., 2000; Xie et al., 2011).

### qRT-PCR validation

Total RNA was extracted with TRIzol (Takara, Tokyo, Japan) and purified with an EasyPure® RNA Purification Kit (TransGen Biotech, Beijing, China). qRT-PCR was performed in three biological replicates using TransScript® Green One-Step qRT-PCR SuperMix (TransGen Biotech, China). Glyceraldehyde-3-phosphate dehydrogenase (GAPDH) was used as an internal reference gene for mRNA (Supplementary Table S5).

## Results

### Fucoxanthin accumulation of *Isochrysis galbana* under different lights

To explore the differences in fucoxanthin accumulation in *I. galbana* under different lights, we detected the fucoxanthin content by HPLC under white and green light at 0 and 5 days. The dry cell weight of *I. galbana* under white light after 5 days (0.18 g/l) was not significantly increased compared to that under green light (0.19 g/l), suggesting that light quality had little effect on biomass accumulation (Figure 1B). The fucoxanthin yield of *I. galbana* in culture, unit dry weight, and unit cell under green light after 5 days (0.71 mg/l, 4.15 mg/g, and 2.04 ug/10^7^cell) increased by 1.25, 1.33, and 1.67 times as those under white light (0.57 mg/l, 3.11 mg/g, and 1.22 ug/10^7^cell), respectively (Figures 1C–E). These results indicate that the fucoxanthin content of *I. galbana* can be significantly increased under green light conditions.

### Landscape of accessible chromatin regions in *Isochrysis galbana* genome

**Figure 2:**
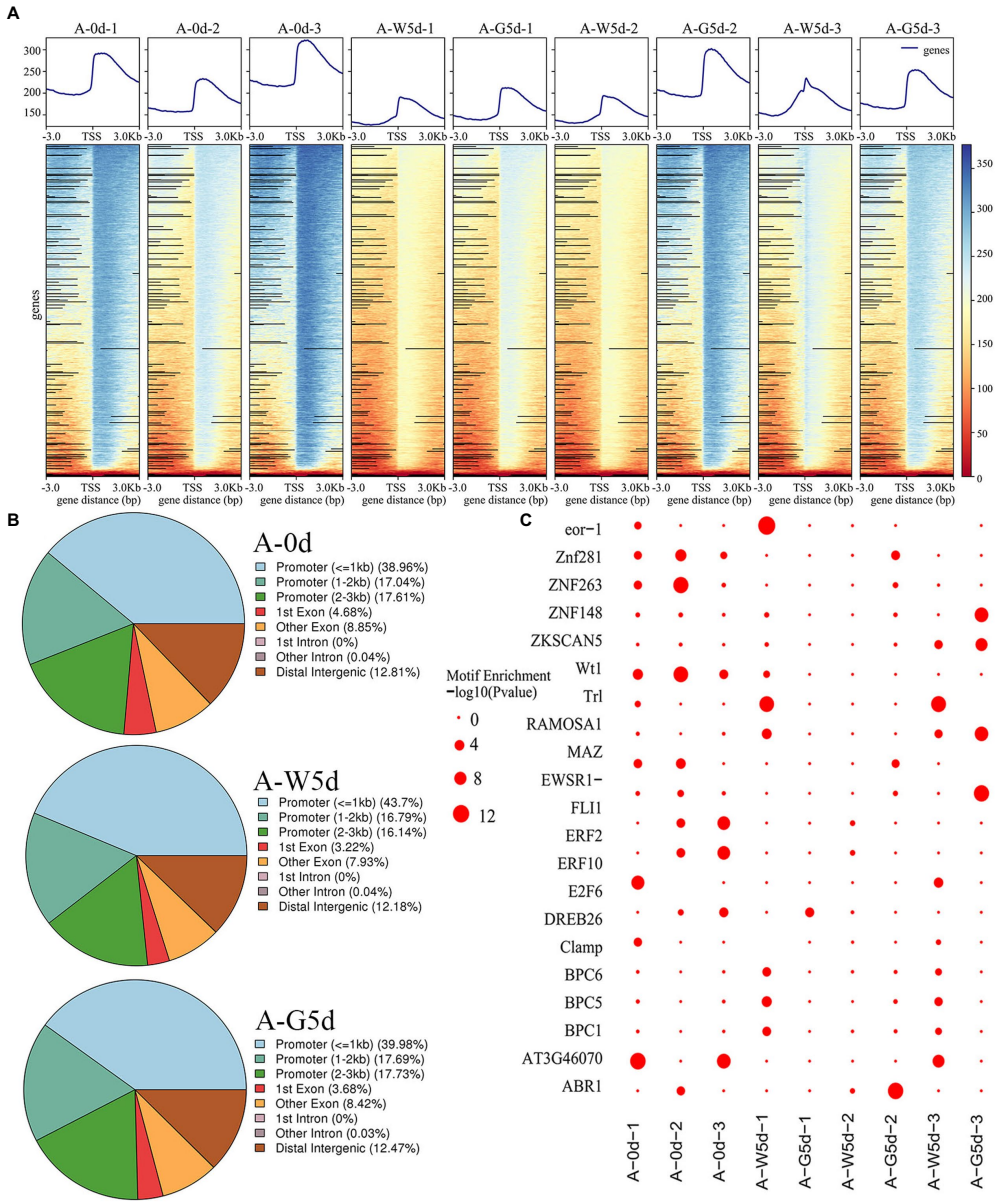
Changes and distribution of chromatin accessibility regions. **(A)** Distribution density map of ATAC data near the TSS. **(B)** Distribution of open chromatin regions accessibility within different genomic regions in A-0d, A-W5d, and A-G5d, respectively. **(C)** Enrichment diagram of transcription factor motifs. Samples were used for ATAC sequencing, namely A-0d, A-W5d, and A-G5d, and each sample was sequenced three times.

A density distribution heatmap showed that genes were enriched around 3 kb upstream and downstream of the transcription start sites (TSSs), suggesting that enriched TSSs at both Day 0 and Day 5 under white or green light exhibited a relatively good signal area (Figure 2A). Pearson correlation analysis showed a high reliability (correlation coefficients, 0.96–0.99) of sampling and data between the biological repeats of A-0d, A-W5d, and A-G5d (Supplementary Figure S1). By peak calling, we obtained 1,330,155 peaks from all samples with the average length of 1,620 bp, and the average percentage of promoter (≤1 kb) and exon obtained after mapping these TSSs of each sample to *I. galbana* genome were 41.33 and 12.17%, respectively (Figure 2B and Supplementary Tables S6, S7). GO and KEGG enrichment analysis of peak-associated genes in promoter regions showed that most of these genes were distributed in metabolic process (GO:0008152), carbohydrate metabolic process (GO:0005975), regulation of transcription (GO:0006355), stimulus response (GO:0050896), carbon metabolism (ko01200), fatty acid metabolism (ko01212), carotenoid biosynthesis (ko00906), and phosphatidylinositol signaling (ko04070) (Supplementary Figures S2, S3). By searching the plant TFs motifs database, we found that a number of TFs belong to ZNF (ZNP148, ZNF263,and ZNF281), ERF (ERF2, ERF10, and ERF104), and BPC (BPC1, BPC5, and BPC6) families within the 20 known motifs, suggesting that they may play a key role in green light culture quality of *I. galbana* (Figure 2C). Additionally, the motifs for ABR, MYB, KLF, RAP, WRKY, and bZIP families were also identified as candidates controlling metabolic regulation responses to green light culture quality of *I. galbana*.

### Identification and enrichment analysis of DARs-associated genes and motifs

**Figure 3:**
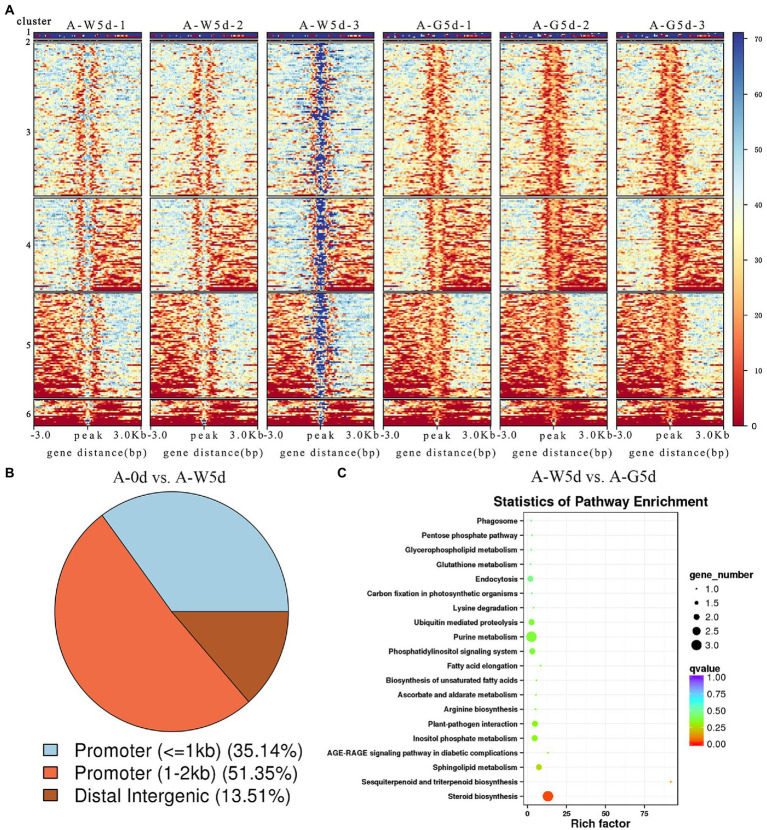
Identification and enrichment analysis of DARs-associated gene and motif. **(A)** Clustering heat map of differential peaks in A-0d, A-W5d, and A-G5d, respectively. **(B)** Annotation classification map of differential peaks in A-0d vs. A-W5d. **(C)** KEGG enrichment of DARs-associated genes in A-W5d vs. A-G5d.

A total of 38, 52, and 264 DARs were identified in A-0d vs. A-W5d, A-0d vs. A-G5d, and A-W5d vs. A-G5d, respectively (Figure 3A). In the comparison of A-0d vs. A-W5d, genome-wide functional regions of DARs were mainly divided into promoter and distal intergenic with 35.14% promoter (≤1 kb) and 51.35% promoter (1–2 kb) (Figure 3B). Functional distribution of DARs-associated genes showed that they were mainly enriched in regulation of transcription (GO:0006355), positive regulation of GTPase activity (GO:0043547), lipid metabolic process (GO:0006629), pentose phosphate pathway (ko00030), carbon fixation in photosynthetic organisms (ko00710), biosynthesis of amino acids (ko01230), and carbon metabolism (ko01200) (Supplementary Figure S4). Additionally, we identified several TFs in DARs-associated motifs, including BPC1 (*value of p* = 4.43242E-08), BPC5 (*value of p* = 1.20238E-07), BPC6 (*value of p* = 2.79864E-08), and RAMOSA1 (*value of p* = 5.53016E-09) (Supplementary Table S8). In the comparison of A-W5d vs. A-G5d, functional regions of DARs were classified into four groups, with the largest number of gene functional elements falling under 1–2 kb promoter (46.25%), followed by ≤1 kb promoter (37.15%), distal intergenic (11.46%), and 2–3 kb promoter (5.14%) (Supplementary Figure S5). GO and KEGG analyses of DARs-associated genes showed that they were mainly enriched in fatty acid biosynthetic process (GO:0006633), signal transduction (GO:0007165), sphingolipid biosynthetic process (GO:0030148), primary metabolic process (GO:0044238), steroid biosynthesis (ko00100), sesquiterpenoid and triterpenoid biosynthesis (ko00909), fatty acid elongation (ko00062) and phosphatidylinositol signaling (ko04070) (Figure 3C; Supplementary Figure S6). DARs-associated motif analysis showed that a number of TFs belong to ZNF, BPC, RAMOSA1, KLF, and Clamp families, which were closely related to *IgDES4* (IZ014017), *IgCYP51A1* (IZ009710), *IgELO3* (IZ000850), *IgPRKG1* (IZ006047), and *IgCPK* (IZ002859).

### Transcriptome profiling and differentially expressed genes

**Figure 4:**
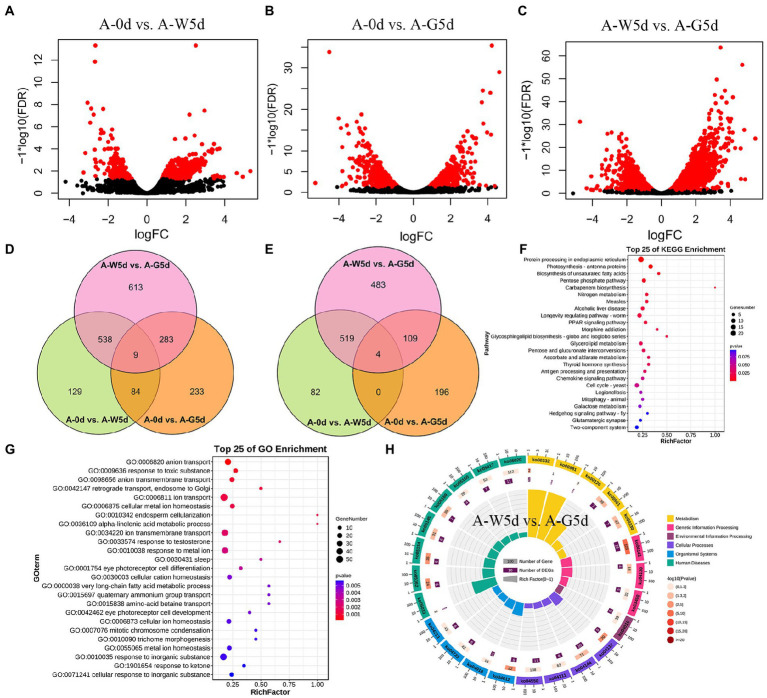
Differential expression and functional enrichment analysis of RNA-seq data. **(A)** Volcano plot of DEGs in A-0d vs. A-W5d. **(B)** Volcano plot of DEGs in A-0d vs. A-G5d. **(C)** Volcano plot of DEGs in A-W5d vs. A-G5d. Blue circles represent up- or downregulated DEGs. **(D)** Venn diagram of all DEGs in A-0d vs. A-W5d, A-0d vs. A-G5d, and A-W5d vs. A-G5d. **(E)** Venn diagram of up regulated DEGs in A-0d vs. A-W5d, A-0d vs. A-G5d, and A-W5d vs. A-G5d. **(F)** Top 25 KEGG enrichment pathways for DEGs in A-0d vs. A-W5d. **(G)** Top 25 GO enrichment pathways for DEGs in A-0d vs. A-G5d. **(H)** KEGG enrichment pathway for DEGs in A-W5d vs. A-G5d.

The number of genes with expression over 6,000 fragments per kilo base of transcript per million fragments mapped varied from 959 to 1,031 in the A-0d, A-W5d, and A-G5d. Pairwise comparison analysis revealed a total of 760 (605 up and 155 down), 609 (309 up and 300 down), and 1,443 (1,115 up and 328 down) DEGs in A-0d vs. A-W5d, A-0d vs. A-G5d, and A-W5d vs. A-G5d, respectively (Figures 4A–C; Supplementary Tables S9–S11). We also identified a total of 6, 12, and 10 TFs in A-0d vs. A-W5d, A-0d vs. A-G5d, and A-W5d vs. A-G5d, respectively. Venn diagram analysis revealed that 129, 233, and 613 DEGs were unique to A-0d vs. A-W5d, A-0d vs. A-G5d, and A-W5d vs. A-G5d, respectively (Figure 4D). Interestingly, the specific number of upregulated DEGs in A-W5d vs. A-G5d (483) was 5.89- and 2.46- fold higher than that in A-0d vs. A-W5d (82) and A-0d vs. A-G5d (196), indicating that green light-mediated culture of *I. galbana* exhibited distinct gene expression profiles after 5 days (Figure 4E). GO and KEGG enrichment analyses showed that these DEGs were significantly classified into biological process (GO:0008150), biosynthetic process (GO:0009058), metabolic process (GO:0008152), biological regulation (GO:0065007), signaling pathways (ko04550), biosynthesis of unsaturated fatty acids (ko01040), terpenoid backbone biosynthesis (ko00900), and starch and sucrose metabolism (ko00500) (Figures 4F,G). Several genes or TFs related to carotenoid metabolism, MAPK signaling pathway, and fructose and mannose metabolism, including *IgMYB98* (IZ007092), *IgPIP5K* (IZ004445), *IgSTK33* (IZ004766), *IgFK* (IZ013898), and *IgP4HA* (IZ004548), were upregulated in A-W5d compared with A-0d (Supplementary Table S12). In the comparison of A-0d vs. A-G5d, some members of the light-harvesting process in energy metabolism showed a higher expression level and were significantly upregulated in A-G5d compared with A-0d, such as *IgLHCA1* (IZ003671), *IgLHCX2* (IZ013939), *IgLHCB4* (IZ000857), and *IgLHCB5* (IZ004582), suggesting that upregulation of these genes by green light may be the key factor leading to the fucoxanthin biosynthesis (Supplementary Table S13). Notable increases in gene expression from the A-W5d to A-G5d included *IgLHCB5* (IZ004582), *IgLHCA1* (IZ003671), *IgMYB98* (IZ007092), *IgNADH* (IZ011907), which are involved in photosynthesis, oxidative phosphorylation, and carotenoids metabolism (Figure 4H; Supplementary Table S14).

### Weighted gene coexpression network analysis involved in Fucoxanthin accumulation

**Figure 5:**
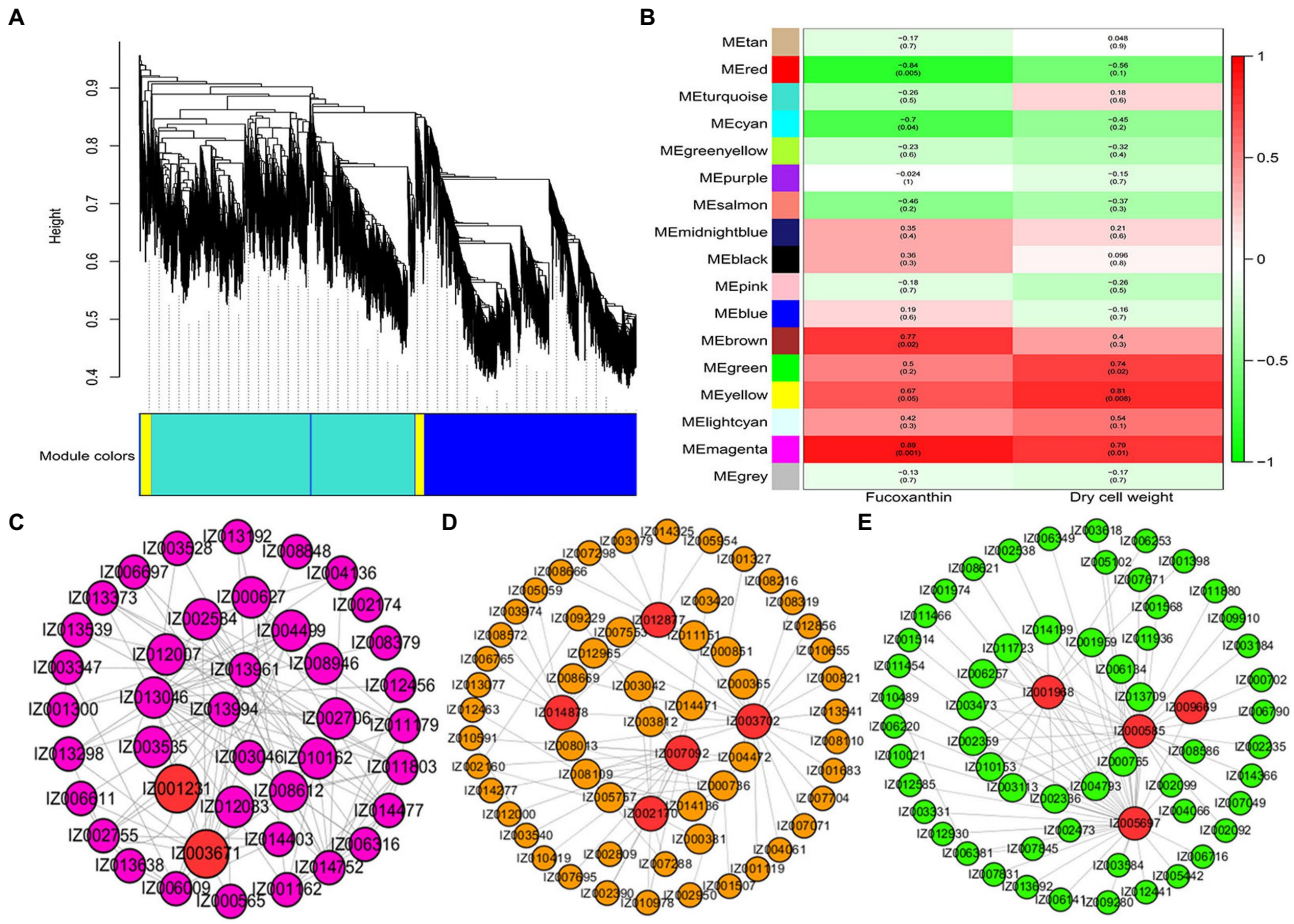
Hub genes related to the content of fucoxanthin and dry cell weight. **(A)** Cluster dendrogram of DEGs for WGCNA analysis. **(B)** Gene modules associated with the content of fucoxanthin and dry cell weight. **(C)** Coexpression network revealing the hub genes related to fucoxanthin and dry cell weight content in the magenta module. **(D)** Coexpression network revealing the hub genes related to fucoxanthin content in the yellow module. **(E)** Coexpression network revealing the hub genes related to fucoxanthin content in the green module.

The soft threshold power of WGCNA was determined to be 13 based on the scale-free model fit analysis (Supplementary Figure S7). A total of 14,029 genes were subjected to WGCNA for identifying associated modules and key genes involved in fucoxanthin accumulation in *I. galbana* (Figure 5A). Sixteen modules were identified, ranging in number of genes from 63 to 4,648; six modules were found to be significant at the defined cut-offs of coefficient value ≥0.6 and *value of p* ≤ 0.05 (Figure 5B). Among them, brown, yellow, and magenta modules were significantly correlated with fucoxanthin content, and green, yellow, and magenta modules were positively correlated with biomass accumulation. Brown and magenta modules were mainly enriched in genes associated with secondary metabolites biosynthesis, photosynthesis, fatty acid metabolism, translation, signal transduction, and terpenoid and polyketide metabolism, including *IgMYB119* (IZ001231), *IgLHCA1* (IZ003671), *IgcrtB* (IZ011432), and *IgMECR* (IZ006116) (Figure 5C; Supplementary Figure S8). Most genes from the yellow module were enriched in starch and sucrose metabolism, carotenoid biosynthesis, fatty acid metabolism, and MAPK signaling pathway, including *IgVDE* (IZ003702), *IgZDS* (IZ006629), *IgfabI* (IZ010243), *IgSCD* (IZ007625), *IgMYB98* (IZ007092), *IgVRG4* (IZ002170), and *IgACD* (IZ008152) (Figure 5D; Supplementary Figure S9). We found that the hub genes *IgPDS* (IZ009969), *IgZEP* (IZ006381), *IgSRP54* (IZ000585) and *IgDNAJB4* (IZ005697) interacted in the green module, which related to carotenoid biosynthesis and genetic information processing (Figure 5E; Supplementary Figure S10). Additionally, several TFs related to carotenoid biosynthesis were also identified in the green module, including *IgMYB2* (IZ001968), *IgMYB33* (IZ005588), *IgMYB42* (IZ009014), and *IgMYB118* (IZ004267).

### Integration analysis of ATAC-seq and RNA-seq

**Figure 6:**
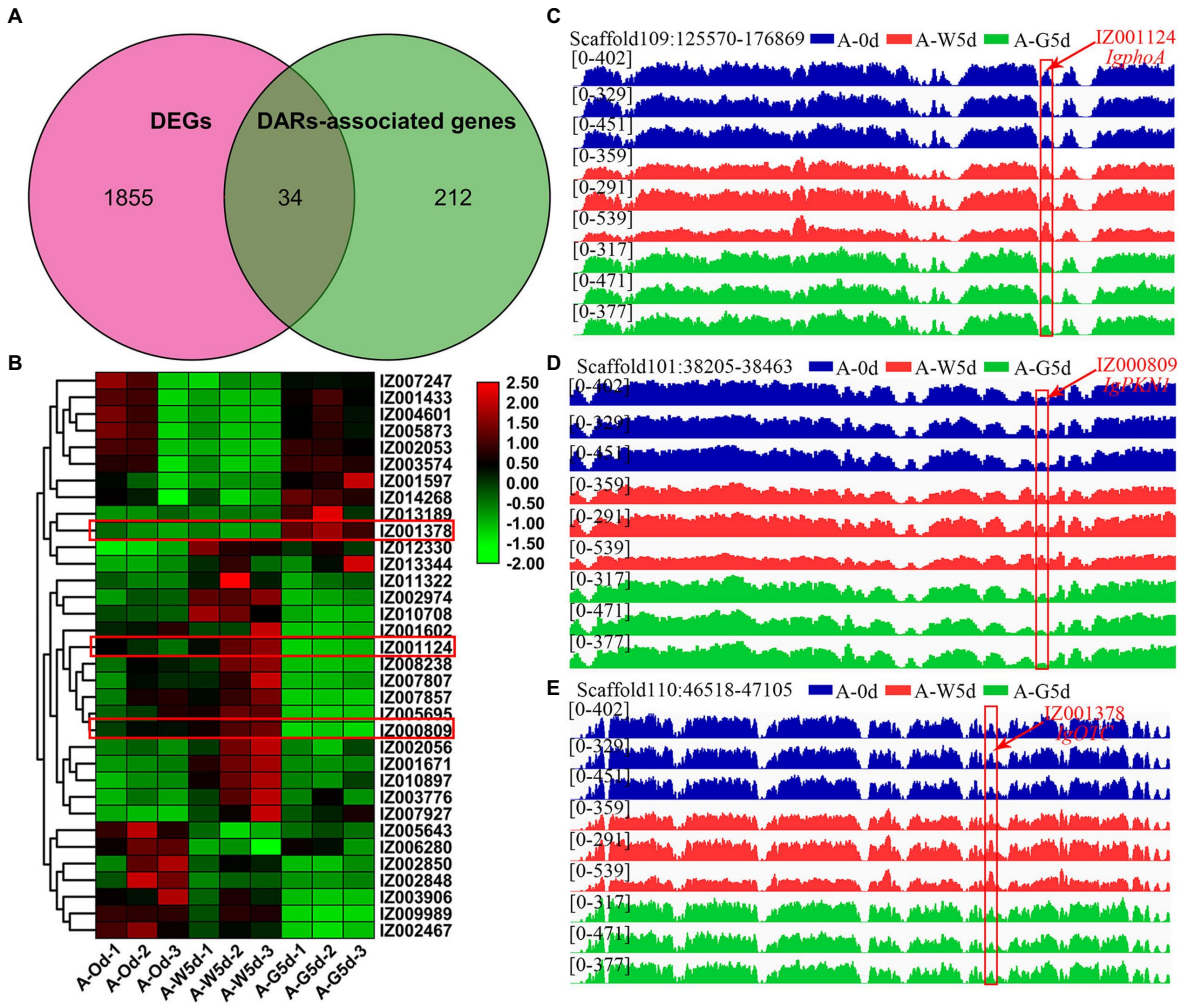
Integration analysis of ATAC-seq and RNA-seq. **(A)** Venn diagram of shared genes between DEGs and DARs-associated genes by RNA-seq and ATAC-seq. **(B)** Heatmap of 34 shared genes visualizing the changes in the expression profiles in A-0d, A-W5d, and A-G5d. **(C)** Overlapping analysis of *IgphoA* gene in DEGs and DARs-associated genes by RNA-seq and ATAC-seq. **(D)** Overlapping analysis of *IgPKN1* gene in DEGs and DARs-associated genes by RNA-seq and ATAC-seq. **(E)** Overlapping analysis of *IgOTC* gene in DEGs and DARs-associated genes by RNA-seq and ATAC-seq.

Association analysis of DARs of ATAC-seq predicted that the open chromatin region mediated by green light caused the change of the ability of the promoter regulatory region, and finally mediated the upregulation of downstream gene expression. Venn diagram analysis revealed that 34 genes were shared by DEGs and DARs-associated genes, whereas 1,855 and 212 genes were unique to DEGs obtained from RNA-seq and DARs-associated genes obtained from ATAC-seq, respectively (Figure 6A). Some of these 34 shared genes exhibited significant expression differences and were involved in cellular processes, amino acid metabolism, MAPK signaling pathway, and signal transduction, including *IgphoA* (IZ001124), *IgPKN1* (IZ000809), *IgOTC* (IZ001378), *IgCOPB2* (IZ002053), *IgABP1* (IZ007807), and *IgRLM1* (IZ010897) (Figure 6B). The 34 DARs-associated genes were analyzed and compared in the open region of chromatin; three genes (*IgphoA*, *IgPKN1*, *IgOTC*) showed obvious changes in the chromatin region in ATAC-seq data. For example, the open chromatin and the differential genes of *IgphoA* and *IgPKN1* were suppressed by green light mediated culture relative to the white light group (Figures 6C,D). *OTC* is a key enzyme gene involved in urea cycle and arginine biosynthesis, participating in the formation of secondary metabolites and amino acids in plants (Zhao et al., 2018). *IgOTC* gene expression increased under green light treatment compared to white light, whereas chromatin opening was affected under both culture cycle and green light treatments (Figure 6E). It is likely that these genes specific for green light play a key role in the process of fucoxanthin biosynthesis in *I. galbana* through a complex regulatory network of multiple metabolic pathways interacting with each other.

### Validation of the candidate genes by real-time quantitative PCR

To further validate the reliability of the gene expression levels, we selected eight candidate genes (*IgphoA*, IZ001124; *IgPKN1*, IZ000809; *IgOTC*, IZ001378; *IgLHCA1*, IZ003671; *IgMYB98*, IZ007092; *IgLHCB4*, IZ000857; *IgDES4*, IZ014017; *IgCYP51A1*, IZ009710) for qRT-PCR analysis. The qRT–PCR results were consistent with the expression levels of RNA-Seq and ATAC-seq (Supplementary Figure S11).

## Discussion

A number of studies have preliminarily explored the regulatory mechanisms of fucoxanthin biosynthesis for the construction of high fucoxanthin content strains (Zhang et al., 2017; Chen et al., 2022; Truong et al., 2022). Recent studies investigated the effects of light treatment on fucoxanthin biosynthesis pathways in *I. galbana* (Chen et al., 2022). However, how the molecular mechanism of fucoxanthin biosynthesis responds to green light-mediated culture for different culture stages remains to be investigated. Although our previous research revealed that green light effectively promoted the accumulation of fucoxanthin, there is little research on chromatin accessibility in the process of transcriptional regulation. In the present study, ATAC-seq was used to analyze the chromatin opening profiles under different light treatments (white and green lights). Integrating ATAC-seq and RNA-seq analyses were performed to identify the key TFs or genes affecting fucoxanthin biosynthesis after green light treatment.

KEGG and GO analyses showed that peak-associated genes involved in carotenoid biosynthesis and photosynthesis-antenna protein formation were significantly enriched, including *IgLHCA1*, *IgLHCA4*, *IgcrtB*, *IgPDS*, *IgZ-ISO*, *IglcyB*, *IgCCD8*, *IgZEP*, and *IgVDE*, indicating that different light qualities play important roles in the regulation of fucoxanthin biosynthesis in *I. galbana*. We found that MYB, bZIP, and WRKY transcription factors, especially the MYB-box, play major roles in fucoxanthin accumulation in *I. galbana* under green light treatment, including *IgMYB1*, *IgMYB2*, *IgMYB33*, *IgMYB42*, *IgMYB98*, *IgMYB118*, and *IgMYB119*. Our previous research found that *IgMYB98* may have a negative regulatory effect on fucoxanthin biosynthesis in *I. galbana* by green light induction (Chen et al., 2022). In kiwifruit, *AcMYB7* activated the promoter of the *AdLCY-β* gene, regulating the biosynthesis of carotenoids and chlorophyll (Ampomah-Dwamena et al., 2019). We further proved that the members of MYB gene family are important regulators for fucoxanthin biosynthesis. DARs-associated motif analysis showed that BPC5 and BPC6 had a strong binding effect on the promoter regions of *IgDES4*, *IgCYP51A1*, *IgELO3*, and *IgPRKG1*, which were closely related to fatty acid biosynthesis, steroid biosynthesis, and signaling pathways. Studies reported that *AtBPC6* was involved in photosynthesis, photoreactions, photosynthetic membranes, and reproductive development processes in petunias (Yu et al., 2022). *AtBPC1*, *AtBPC2*, *AtBPC4*, and *AtBPC6* were involved in the regulation of plant seedling growth, development, and stress responses in *Arabidopsis thaliana* (Mu, 2016). A linear relationship between the synthesis of pigment and fatty acids has been reported, and the upregulation of genes related to fatty acid metabolism was partly responsible for the large accumulation of pigment in *Haematococcus pluvialis* (Zhekisheva et al., 2002; Saha et al., 2013; Cheng et al., 2017). We speculated that BPC6 may regulate *IgDES4* and *IgELO3* to participate in lipid metabolism, resulting in a higher fucoxanthin content in *I. galbana* under green light treatment.

To better understand the regulatory mechanisms of fucoxanthin accumulation, WGCNA was performed to reveal the candidate genes involved in the fucoxanthin biosynthesis. *IgcrtB*, *IgVDE*, *IgZDS*, *IgPDS*, and *IgZEP* genes from green and yellow modules were significantly correlated with fucoxanthin content. For example, the high expression level of *PtVDE* gene played a critical regulatory role in fucoxanthin biosynthesis of *Phaeodactylum tricornutum* (Yang and Wei, 2020). The upregulation of the *PtZDS* gene resulted in the strongest synthesis ability of fucoxanthin in *P. tricornutum* under the treatment of 100 μmol/l MeJA (Zhang et al., 2017). We suggested that the expression of genes upstream of the carotenoid biosynthetic pathway plays a crucial role in the downstream of fucoxanthin accumulation. *LHCA1* is a chlorophyll a/b-binding protein that plays a key role in plant photosynthesis (Zou et al., 2014). *LHCB*5 and *LHCB6* play unique roles in plant development, thylakoid organization, and PSII photo-protection (Gao et al., 2009). Interestingly, we found that the hub genes *IgLHCA1* and *IgLHCB5* showed higher expression levels and were significantly upregulated in A-G5d compared with A-0d and A-G5d, indicating that the high expression or upregulation of these genes can affect the fucoxanthin biosynthesis by regulating the photosynthesis-antenna protein pathway. An integrated analysis of ATAC-seq and RNA-seq showed the importance of *IgphoA*, *IgPKN1*, *IgOTC*, and fucoxanthin biosynthesis in *I. galbana* under green light treatment. The *phoA* gene participated in the encoding and synthesis of alkaline phosphatase, which has a signal peptide, playing a key role in cofactor biosynthesis (Kikuchi et al., 1981). Algae alkaline phosphatase (AP) belonging to an atypical type AP (PhoA(aty)) could utilize marine phytoplankton to scavenge phosphorus from dissolved organic phosphorus (Lin et al., 2015). In addition to utilizing phosphorus nutrients, AP also inhibits pigment synthesis, photosynthesis, fatty acid synthesis, and cell division, maintains the metabolic homeostasis, and prevents premature cell division (Zhang et al., 2021). We speculated that alkaline phosphatase is the most important organophosphorus hydrolase by releasing inorganic phosphorus for the growth and metabolism of algae, which may promote the synthesis of secondary metabolites to a certain extent. *PKN1* is an important regulator of growth and metabolism, which participates in signal transduction, the cell cycle, cell growth, and gene expression (He et al., 2015). The *PKNs* genes in *Arabidopsis* and *Pharbitis* had been found to play a role in cell division, regulating the differentiation of shoots, leaves and roots (Kobayashi et al., 2000). The downregulation of *IgPKN1* under green light may partially inhibit other physiological metabolic processes, promoting the accumulation of fucoxanthin in *I. galbana* cells. These findings will facilitate in-depth understanding the molecular regulation mechanisms of fucoxanthin in *I. galbana* and its role in response to green light regulation, providing technical support for the construction of high fucoxanthin content strains.

## Data availability statement

mRNA and ATAC sequencing data have been deposited at the National Genomics Data Center, Beijing Institute of Genomics, Chinese Academy of Sciences, under BioProject accession number CRA008916 and CRA008876 (https://ngdc.cncb.ac.cn/gsa/). The names of the repository/repositories and accession number(s) can be found in the article/Supplementary material.

## Author contributions

YC and TX designed and coordinated the entire project. YH, XZ, JingC, YX, and TC performed the collection and processing of samples. TX, DC, HL, XC, and JiannanC performed the omics analysis. TX, HL, and YC participated in manuscript writing and revision. All authors have read and approved the final manuscript.

## Funding

This work was supported by the National Natural Science Foundation of China (Grant No. 42006087) and China Agriculture Research System of MOF and MARA (Grant No. CARA-170501).

## Conflict of interest

The authors declare that the research was conducted in the absence of any commercial or financial relationships that could be construed as a potential conflict of interest.

## Publisher’s note

All claims expressed in this article are solely those of the authors and do not necessarily represent those of their affiliated organizations, or those of the publisher, the editors and the reviewers. Any product that may be evaluated in this article, or claim that may be made by its manufacturer, is not guaranteed or endorsed by the publisher.

## Supplementary material

The Supplementary material for this article can be found online at: https://www.frontiersin.org/articles/10.3389/fmicb.2023.1101681/full#supplementary-material
