# Supplementary material for: Combined analysis of chromatin accessibility and gene expression profiles provide insight into Fucoxanthin biosynthesis in Isochrysis galbana under green light
Source: Front Microbiol. 2023 Feb 10;14:1101681. doi: 10.3389/fmicb.2023.1101681 (PMC9950639; doi:10.3389/fmicb.2023.1101681)
Supplement: Supplementary file 1 [file Data_Sheet_1.zip › Supplementary Figures.doc]

**Supplementary Figures**

**Figure S1** Clustering heatmap of sample correlation by ATAC-seq.

A	B

C

**Figure S2** GO annotation of peak region related genes in A-0d (A), A-W5d (B), and A-G5d (C).

A	B

C

**Figure S3** KEGG annotation of peak region related genes in A-0d (A), A-W5d (B), and A-G5d (C).

A	B

**Figure S4** GO (A) and KEGG (B) enrichment analysis of DARs-associated in A-0d vs. A-W5d.

**Figure S5** Annotation classification map of differential peak in A-W5d vs. A-G5d.

**Figure S6** GO enrichment analysis of DARs-associated in A-W5d vs. A-G5d.

**Figure S7** Soft Threshold.

A	B

**Figure S8** KEGG enrichment analysis of genes from brown (A) and magenta (B) modules.

**Figure S9** KEGG enrichment analysis of genes from yellow module.

**Figure S10** KEGG enrichment analysis of genes from green module.

**Figure S11** Real-time quantitative RT-qPCR confirmation of *IgphoA*, *IgPKN1*, *IgOTC*, *IgLHCA1*, *IgMYB98*, *IgLHCB4*, *IgDES4*, and *IgCYP51A1*. Relative gene expressions were analyzed using the 2^−ΔΔCt^ method. Experiments were performed in triplicate. Error bars indicate standard deviation.
