# Supplementary material for: Combined analysis of chromatin accessibility and gene expression profiles provide insight into Fucoxanthin biosynthesis in Isochrysis galbana under green light
Source: Front Microbiol. 2023 Feb 10;14:1101681. doi: 10.3389/fmicb.2023.1101681 (PMC9950639; doi:10.3389/fmicb.2023.1101681)
Supplement: Supplementary file 1 [file Data_Sheet_1.zip › Supplementary Tables 1-8 and 12-14.doc]

**Supplementary Tables**

Table S1 Statistics of sample ATAC-seq data

| Sample | Repeat | Clean_Reads | GC(%) | N(%) | Q20(%) | Q30(%) |
| --- | --- | --- | --- | --- | --- | --- |
| A-0d | A-0d-1 | 148,161,732 | 56.47 | 0 | 97.57 | 94.15 |
|  | A-0d-2 | 109,505,941 | 57.15 | 0 | 97.47 | 93.97 |
|  | A-0d-3 | 156,664,695 | 56.78 | 0 | 97.56 | 94.19 |
| A-W5d | A-W5d-1 | 119,787,694 | 55.25 | 0 | 97.55 | 94.18 |
|  | A-W5d-2 | 113,798,125 | 56.03 | 0 | 97.45 | 93.99 |
|  | A-W5d-3 | 120,995,709 | 55.11 | 0 | 96.71 | 92.35 |
| A-G5d | A-G5d-1 | 116,544,435 | 56.73 | 0 | 97.37 | 93.32 |
|  | A-G5d-2 | 147,035,752 | 56.64 | 0 | 96.99 | 92.71 |
|  | A-G5d-3 | 125,447,486 | 56.25 | 0 | 97.19 | 92.93 |

Table S2 Statistical table showing the alignment of each sample with the reference genome by ATAC-seq

| Sample | Repeat | Total_Reads | Mapped_Reads | Uniq_Mapped_Reads | MT(%) | Duplication(%) |
| --- | --- | --- | --- | --- | --- | --- |
| A-0d | A-0d-1 | 148161732 | 130730514(88.24%) | 98203558(66.28%) | 0 | 18.81 |
|  | A-0d-2 | 109505941 | 100631271(91.90%) | 75559853(69%) | 0 | 18.58 |
|  | A-0d-3 | 156664695 | 140906787(89.94%) | 105812550(67.54%) | 0 | 18.74 |
| A-W5d | A-W5d-1 | 119787694 | 95436850(79.67%) | 71804253(59.94%) | 0 | 22.38 |
|  | A-W5d-2 | 113798125 | 96855667(85.11%) | 72627810(63.82%) | 0 | 22.54 |
|  | A-W5d-3 | 120995709 | 96600381(79.84%) | 72718255(60.09%) | 0 | 16.17 |
| A-G5d | A-G5d-1 | 116544435 | 103480777(88.79%) | 77386183(66.4%) | 0 | 22.42 |
|  | A-G5d-2 | 147035752 | 128169882(87.17%) | 95485819(64.94%) | 0 | 17.5 |
|  | A-G5d-3 | 125447486 | 106762871(85.11%) | 79548295(63.41%) | 0 | 15.37 |

Table S3 Statistics of sample RNA-seq data

| Sample | Repeat | Total_reads | Total_bases | Q30 |
| --- | --- | --- | --- | --- |
| R-0d | R-0d-1 | 82,271,478 | 12,340,721,700 | 90.82% |
|  | R-0d-2 | 85,417,812 | 12,812,671,800 | 92.01% |
|  | R-0d-3 | 92,750,238 | 13,912,535,700 | 93.02% |
| R-W5d | R-W5d-1 | 109,665,534 | 16,449,830,100 | 92.29% |
|  | R-W5d-2 | 93,155,890 | 13,973,383,500 | 89.76% |
|  | R-W5d-3 | 83,308,448 | 12,496,267,200 | 90.48% |
| R-G5d | R-G5d-1 | 82,685,654 | 12,402,848,100 | 91.14% |
|  | R-G5d-2 | 83,617,610 | 12,542,641,500 | 92.83% |
|  | R-G5d-3 | 65,585,916 | 9,837,887,400 | 90.44% |

Table S4 Statistical table showing the alignment of each sample with the reference genome by RNA-seq

| Sample | Repeat | Total_reads | Unique_mapped | Multiple_mapped |
| --- | --- | --- | --- | --- |
| R-0d | R-0d-1 | 41,135,739 | 93.56 | 1.42 |
|  | R-0d-2 | 42,708,906 | 93.48 | 1.45 |
|  | R-0d-3 | 46,375,119 | 93.63 | 1.61 |
| R-W5d | R-W5d-1 | 54,832,767 | 92.76 | 1.7 |
|  | R-W5d-2 | 46,577,945 | 93.34 | 1.68 |
|  | R-W5d-3 | 41,654,224 | 92.60 | 1.93 |
| R-G5d | R-G5d-1 | 41,342,827 | 93.83 | 1.52 |
|  | R-G5d-2 | 41,808,805 | 93.61 | 1.68 |
|  | R-G5d-3 | 32,792,958 | 93.35 | 1.6 |

Table S5 Primers for genes validated by quantitative real-time PCR

| Genes | Sequences 5’-3’ |
| --- | --- |
| *GDAPH-F* | CCGTTCATCACCGTTGACTACA |
| *GDAPH-R* | AACAGTCACAGGCTTTTCACCAA |
| *IgphoA-F* | ATGATGCGTGCTTCTGTG |
| *IgphoA-R* | TCAGAATGCAAAGCTGAGAAG |
| *IgPKN1-F* | ATGGCGCCTGAGCTGATGACAC |
| *IgPKN1-R* | CTATGCGCTGGCCGCCAC |
| *IgOTC-F* | ATGCTCTGTTCTCTCCTTTTCTCCTC |
| *IgOTC-R* | TCAAGGACGTGCAGCATTGG |
| *IgLHCA1-F* | ATGCTCTCGCTCTACTCTGCTGTTC |
| *IgLHCA1-R* | TTAGAGGCCTGAAGCGGCGTC |
| *IgMYB98-F* | ATGGCTGAGCTTGACCATTCAGG |
| *IgMYB98-R* | TCAAGCCGTCACCCGTGC |
| *IgLHCB4-F* | ATGTGCCACTTGTCGACTTC |
| *IgLHCB4-R* | TTAGAACCCGAAGGTCGACC |
| *IgDES4-F* | ATGGGCAAGGGCGGCTCC |
| *IgDES4-R* | TCATTCGTCCTTTGTCGTCATGCGCTTG |
| *IgCYP51A1-F* | ATGTGCGACGTGTCCGAC |
| *IgCYP51A1-R* | CTACGTAGCTGCAACACGAGTC |

Table S6 Statistics of calling peak number

| Sample | Repeat | Number | 1st_  quarter_length | Median | Mean | 3rd_  quarter_length | Max_length |
| --- | --- | --- | --- | --- | --- | --- | --- |
| A-0d | A-0d-1 | 160310 | 833 | 1734 | 1867 | 2691 | 6935 |
|  | A-0d-2 | 150947 | 682 | 1463 | 1619 | 2348 | 6064 |
|  | A-0d-3 | 157033 | 719 | 1536 | 1688 | 2441 | 6431 |
| A-W5d | A-W5d-1 | 142338 | 596 | 1280 | 1476 | 2173 | 6097 |
|  | A-W5d-2 | 149918 | 646 | 1413 | 1587 | 2317 | 6797 |
|  | A-W5d-3 | 140857 | 699 | 1434 | 1611 | 2329 | 6740 |
| A-G5d | A-G5d-1 | 142769 | 644 | 1382 | 1559 | 2263 | 6076 |
|  | A-G5d-2 | 143068 | 683 | 1428 | 1593 | 2292 | 6428 |
|  | A-G5d-3 | 142915 | 668 | 1418 | 1582 | 2291 | 6350 |

Table S7 Reads distribution of gene functional components

| Sample | Repeat | Promoter (<=1kb)(%) | Promoter (1-2kb)(%) | Promoter (2-3kb)(%) | Exon(%) | Intron(%) | Distal Intergenic(%) |
| --- | --- | --- | --- | --- | --- | --- | --- |
| A-0d | A-0d-1 | 42.54 | 14.66 | 16.01 | 14.35 | 0.03 | 12.41 |
|  | A-0d-2 | 38.57 | 17.98 | 17.92 | 12.47 | 0.04 | 13.01 |
|  | A-0d-3 | 38.96 | 17.04 | 17.61 | 13.54 | 0.04 | 12.81 |
| A-W5d | A-W5d-1 | 42.2 | 17.96 | 16.92 | 10.74 | 0.04 | 12.15 |
|  | A-W5d-2 | 43.7 | 16.79 | 16.14 | 11.15 | 0.04 | 12.18 |
|  | A-W5d-3 | 50.72 | 15.38 | 13.74 | 9.36 | 0.04 | 10.77 |
| A-G5d | A-G5d-1 | 39.98 | 17.69 | 17.73 | 12.1 | 0.04 | 12.47 |
|  | A-G5d-2 | 37.64 | 18.28 | 18.22 | 13.08 | 0.03 | 12.75 |
|  | A-G5d-3 | 37.65 | 18.39 | 18.34 | 12.76 | 0.04 | 12.82 |

Table S8 Motif annotation results

| Target ID | factor | Optimal offset | p-value | E-value | q-value | Overlap | Orientation |
| --- | --- | --- | --- | --- | --- | --- | --- |
| MA1403.1 | BPC5 | 1 | 2.69E-10 | 4.43E-07 | 8.81E-07 | 29 | + |
| MA1404.1 | BPC1 | -2 | 8.00E-09 | 1.32E-05 | 1.31E-05 | 24 | + |
| MA1402.1 | BPC6 | -8 | 2.98E-08 | 4.90E-05 | 3.25E-05 | 21 | - |
| MA1416.1 | RAMOSA1 | -12 | 2.48E-06 | 0.0041 | 0.002 | 14 | + |

Table S12 Annotation for up- regulated DEGs in A-0d vs. A-W5d.

| ID | Name | KO number | ID | Name | KO number |
| --- | --- | --- | --- | --- | --- |
| IZ011353 |  | K19953 | IZ004521 | PDIA4 | K09582 |
| IZ008230 | RPGR | K19607 | IZ004180 |  | K09553,K16365 |
| IZ001387 | CLEC16A | K19513 | IZ001510 |  | K09553 |
| IZ002955 | ANO3 | K19498,K19499,K19503 | IZ009094 |  | K09553 |
| IZ002027 | DNAJC22 | K19370 | IZ011569 |  | K09536 |
| IZ005208 |  | K19355 | IZ001692 |  | K09527 |
| IZ006795 |  | K19232 | IZ009997 |  | K09527 |
| IZ003385 |  | K19232 | IZ007092 |  | K09422 |
| IZ014467 |  | K19232 | IZ009065 | SPNS3 | K09008,K12890 |
| IZ013670 | ANP32B | K18647 | IZ007245 | YNEE | K08994 |
| IZ002323 |  | K18442 | IZ009351 |  | K08869 |
| IZ011670 |  | K17981 | IZ004324 |  | K08853 |
| IZ014527 |  | K17964 | IZ011830 |  | K08853 |
| IZ012400 |  | K17865,K18532 | IZ004766 | STK33 | K08813 |
| IZ000459 | WDR81 | K17601 | IZ002518 |  | K08770 |
| IZ003003 | SCAT | K17600 | IZ010735 | RCE1 | K08658 |
| IZ001180 | URI1 | K17560 | IZ013845 |  | K08494 |
| IZ007349 |  | K17545 | IZ005329 |  | K08493 |
| IZ010833 |  | K17545 | IZ013839 | STX5 | K08490 |
| IZ007835 |  | K17545 | IZ004390 |  | K08489 |
| IZ002167 |  | K17086 | IZ012578 |  | K08288 |
| IZ006707 |  | K16675 | IZ003012 | PTK2 | K08286 |
| IZ003248 | LRRC49 | K16606 | IZ007114 | CPI1 | K08246 |
| IZ007234 | RPGRIP1 | K16512,K16550 | IZ003697 |  | K07904,K07905 |
| IZ012216 | CEP76 | K16457 | IZ013600 | RAB8 | K07901 |
| IZ008053 | PPP4R1 | K15424 | IZ007883 |  | K07752 |
| IZ003323 | PGUG_04898 | K15289 | IZ012273 |  | K07304 |
| IZ002006 |  | K15283 | IZ001685 | SCO1 | K07152 |
| IZ014248 |  | K15283 | IZ010809 | YBEQ | K07126 |
| IZ003971 | SLC35A2 | K15272 | IZ009366 | FG10381.1 | K06683 |
| IZ002426 |  | K15272 | IZ003845 | SPG1 | K06682,K07976 |
| IZ005770 | RTF1 | K15178 | IZ007981 | OSI_21267 | K05941 |
| IZ012370 | SUPT4H1 | K15171 | IZ000691 | PLCE1 | K05860 |
| IZ003510 |  | K15153 | IZ000941 |  | K05747 |
| IZ005241 |  | K15109 | IZ002250 | KCO1 | K05389 |
| IZ007607 |  | K15105 | IZ000886 | SETD6 | K05302 |
| IZ001143 |  | K15085 | IZ000165 |  | K04874 |
| IZ006904 |  | K15015 | IZ004033 | E2F | K04682,K06620 |
| IZ005789 |  | K14457 | IZ002676 |  | K04648,K10392,K11498 |
| IZ002008 | SLC13A5 | K14445 | IZ003507 | PUC | K04459 |
| IZ014251 |  | K14439 | IZ009213 | CTPA | K03797 |
| IZ010642 |  | K14439 | IZ003819 | PPIF | K03767,K09565 |
| IZ002190 |  | K14439 | IZ000031 | PROP | K03762,K12226 |
| IZ000644 |  | K14439 | IZ013900 | STX6 | K03691,K08498,K08500 |
| IZ001579 |  | K14439 | IZ013637 |  | K03654,K10900 |
| IZ010858 |  | K14439 | IZ002881 | TOLA | K03646 |
| IZ014899 |  | K14427 | IZ004663 | LEPA | K03596 |
| IZ007954 | ZDHHC15 | K14423,K18932,K20028 | IZ004632 | POLI | K03510 |
| IZ007817 | HBS1L | K14416 | IZ000620 | HSP70 | K03283 |
| IZ005165 |  | K14191 | IZ008489 | TCEA1 | K03145,K04533,K15169 |
| IZ010920 | SEL1L | K14026 | IZ000172 |  | K03128 |
| IZ012384 | PUX3 | K14012 | IZ005385 |  | K03118,K03407,K03529,K03572,K07332,K09746 |
| IZ005655 | MUC7 | K13909 | IZ006532 | FTSY | K03110 |
| IZ005335 |  | K13862 | IZ012386 | INPP5D | K03084,K15909 |
| IZ005448 | DAGLB | K13806 | IZ000084 | MKS1 | K03030,K19332 |
| IZ011337 |  | K13806 | IZ005884 | RPLV | K02890 |
| IZ000987 | ABHD6 | K13700 | IZ002202 | FUCU | K02431 |
| IZ000903 |  | K13507 | IZ005717 |  | K02206 |
| IZ010706 |  | K13447 | IZ002716 | GTPBP8 | K02154,K03978 |
| IZ005218 | CPK20 | K13412 | IZ012082 |  | K01999 |
| IZ014731 |  | K13412 | IZ000291 | ACSL4 | K01897 |
| IZ008256 |  | K13348 | IZ011736 |  | K01886 |
| IZ005507 |  | K13348 | IZ009918 |  | K01872 |
| IZ014894 | WPRA | K13274,K14645 | IZ009191 | GPMB | K01834,K15634 |
| IZ000647 | GPATCH1 | K13123 | IZ005971 | MGL1 | K01739,K01761 |
| IZ010502 | RBMX2 | K13107 | IZ014169 | ENO1 | K01689 |
| IZ010768 |  | K13099 | IZ010070 | ATP13A1 | K01537,K14950 |
| IZ009236 | TXNL4A | K12859 | IZ012586 | ECA2 | K01537,K05853 |
| IZ005885 |  | K12855 | IZ009318 |  | K01530,K14802 |
| IZ000784 | OSI_06346 | K12823 | IZ014363 |  | K01519,K08869 |
| IZ009640 |  | K12816 | IZ002483 |  | K01505,K05396 |
| IZ014812 | MYO10 | K12559 | IZ012121 |  | K01456 |
| IZ008981 |  | K12492 | IZ004238 | IDE | K01408 |
| IZ012474 |  | K12472 | IZ009666 |  | K01408 |
| IZ013202 |  | K12391 | IZ001710 | CP3 | K01365,K01371,K08570 |
| IZ004043 |  | K11978 | IZ008835 | PTRB | K01322,K01354 |
| IZ012193 |  | K11887 | IZ014565 |  | K01267 |
| IZ001980 |  | K11840,K11853 | IZ003291 | PIP | K01259 |
| IZ013740 |  | K11778 | IZ012470 |  | K01193 |
| IZ008412 | FOLC | K11754 | IZ006156 |  | K01179 |
| IZ001282 |  | K11744 | IZ003525 |  | K01130,K01134,K01135,K01136,K01137,K01138 |
| IZ013178 | PGGT1B | K11713 | IZ012185 |  | K01113 |
| IZ012281 |  | K11654 | IZ005176 | ABAT | K01099,K13524,K14005 |
| IZ010044 |  | K11494 | IZ011235 | SYNJ1 | K01099 |
| IZ014208 | CHM | K11450,K17255,K17878 | IZ003450 |  | K01082 |
| IZ014314 | LID | K11446 | IZ008109 | PTH | K01056 |
| IZ002472 | G9A | K11420 | IZ000799 | FG10339.1 | K01053 |
| IZ004750 |  | K11153 | IZ008730 | UGP1 | K00963,K01835,K02987 |
| IZ003005 |  | K10901 | IZ012883 |  | K00953 |
| IZ012461 | FANCI | K10895 | IZ010224 | FAM18A | K00948 |
| IZ000404 | ALKBH6 | K10768 | IZ007305 | PROB | K00931 |
| IZ000482 |  | K10728 | IZ008164 | ITPKA | K00911 |
| IZ009108 |  | K10712 | IZ001242 | ADRBK1 | K00910 |
| IZ002906 | SAE2 | K10685 | IZ002520 | PDK | K00898 |
| IZ003192 | PTCHD2 | K10649,K18730 | IZ004445 |  | K00889,K00920 |
| IZ012338 | SYVN1 | K10601,K17262 | IZ003013 | RBSK | K00852 |
| IZ011423 | HERC2 | K10595 | IZ013898 | SCRK | K00845,K00847,K00885 |
| IZ008447 | UBE3B | K10588 | IZ011819 |  | K00771 |
| IZ008378 | HECTD2 | K10587,K10614,K12232 | IZ002890 |  | K00757 |
| IZ000487 |  | K10461,K10467 | IZ001340 | SCLAV_2631 | K00729 |
| IZ008699 | MAPRE1 | K10436 | IZ000900 | OCAR_7038 | K00641 |
| IZ001352 | CLIP1 | K10421 | IZ001378 | OTC | K00611 |
| IZ006505 |  | K10408,K10413 | IZ004845 |  | K00592 |
| IZ000215 |  | K10408 | IZ007051 | DCM | K00571,K03497 |
| IZ008485 |  | K10393 | IZ005432 | CYP51 | K00493,K05917,K14338,K15470 |
| IZ006173 | PLEC | K10388,K19827 | IZ004548 |  | K00472 |
| IZ005010 | MYO6 | K10358 | IZ008413 | PGUG_05820 | K00471 |
| IZ013960 | MYO2 | K10357 | IZ013908 | FG03227.1 | K00344,K01178 |
| IZ006170 |  | K10260 | IZ011510 |  | K00329,K00356 |
| IZ013437 | EI24 | K10134 | IZ010434 | P5CS | K00147,K12657 |
| IZ001531 |  | K09931 | IZ002763 | PGUG_02249 | K00147 |
| IZ003577 | GYLTL1B | K09668 | IZ003091 | DLD | K00102,K00104,K03777 |
| IZ005798 |  | K09597 | IZ004225 | UGD2 | K00012 |

Table S13 Annotation for up- regulated DEGs in A-0d vs. A-G5d.

| geneID | Name | KO number | geneID | Name | KO number |
| --- | --- | --- | --- | --- | --- |
| IZ013233 |  | K19758 | IZ014202 |  | K04412 |
| IZ012330 |  | K19232 | IZ003177 | DNAK | K04043 |
| IZ000795 | BPHL | K18399 | IZ007528 |  | K03979 |
| IZ008527 | NCA2 | K18158 | IZ013158 |  | K03885,K17871 |
| IZ013247 | AFE_2601 | K17218 | IZ011907 | NDH | K03885 |
| IZ006009 | ANKHD1 | K16726 | IZ009751 | PGUG_04221 | K03809,K14015 |
| IZ005377 |  | K15777 | IZ006133 | HSP104 | K03695 |
| IZ010829 |  | K15642,K16128 | IZ004561 |  | K03676 |
| IZ011880 |  | K15634 | IZ009182 |  | K03676 |
| IZ003832 | FG01886.1 | K15559 | IZ007400 |  | K03676 |
| IZ010830 |  | K15320 | IZ006213 | SMPB | K03664 |
| IZ011452 |  | K15108 | IZ005545 | TOLA | K03646 |
| IZ006559 |  | K15083 | IZ011938 | CDC20.1 | K03363,K15028 |
| IZ009579 |  | K14640 | IZ005923 |  | K03354 |
| IZ005838 |  | K14640 | IZ014037 |  | K03283 |
| IZ009661 |  | K14640 | IZ014038 |  | K03283 |
| IZ003433 |  | K14457 | IZ004588 | TOF1 | K03155,K10997 |
| IZ010260 | CCRA | K14446,K14469,K17829 | IZ007484 | PSBP3 | K02717 |
| IZ000281 |  | K14439 | IZ005302 | PETF1 | K02639 |
| IZ001996 |  | K14439 | IZ014752 | MCM6 | K02542 |
| IZ008256 |  | K13348 | IZ002639 | POLA | K02335,K03469,K06966 |
| IZ013928 | MANF | K13190 | IZ004708 | MCM4 | K02212 |
| IZ000594 |  | K12608 | IZ000453 | MCM7 | K02210 |
| IZ005195 | HEXB | K12373 | IZ007882 |  | K02150 |
| IZ012458 | OSI_18234 | K11271 | IZ004719 |  | K01893 |
| IZ004337 | PDS5B | K11267 | IZ013593 |  | K01889 |
| IZ006341 |  | K11153,K11161 | IZ009491 | CYP9 | K01802 |
| IZ012461 | FANCI | K10895 | IZ004246 |  | K01792,K05275 |
| IZ008963 | RHL2 | K10878 | IZ012573 |  | K01792,K05275 |
| IZ010928 | HERC2 | K10595 | IZ004959 | YEAD | K01792 |
| IZ014221 |  | K10563 | IZ009976 |  | K01689 |
| IZ013660 | FADS1 | K10224,K10225,K10226 | IZ004702 | PHR | K01669 |
| IZ007080 | LMAN2 | K10082,K10083,K14792 | IZ000855 | PPA2 | K01507,K06094,K11726 |
| IZ011233 | CDC37 | K09554 | IZ013384 | ASPG | K01424,K13278 |
| IZ005928 |  | K09516,K09835 | IZ003688 | NUC | K01174 |
| IZ009267 | HSP90B | K09487 | IZ008436 | PHOB | K01077 |
| IZ009655 | HSF2 | K09414,K09415 | IZ009260 |  | K00873 |
| IZ004582 | LHCB5 | K08916 | IZ008671 |  | K00861 |
| IZ000857 | LHCB5 | K08916 | IZ003254 |  | K00833,K12256,K16871 |
| IZ003671 | LHCX2 | K08907 | IZ004498 | GSTCH1 | K00799,K03599 |
| IZ003673 | LHCX2 | K08907 | IZ002003 | FGSG_01294 | K00799 |
| IZ013939 | LHCX2 | K08907 | IZ010508 |  | K00799 |
| IZ011173 |  | K08907 | IZ013046 |  | K00766 |
| IZ005439 |  | K08869 | IZ009410 | PGUG_00803 | K00706,K11000 |
| IZ011835 |  | K08869 | IZ005844 | DLAT | K00627 |
| IZ000624 |  | K08857 | IZ005475 | CPT2 | K00624,K08766 |
| IZ002012 |  | K08286 | IZ004845 |  | K00592 |
| IZ014476 |  | K07942 | IZ006593 | TPM | K00569 |
| IZ005786 |  | K07767 | IZ008375 | NRDA | K00525 |
| IZ000859 | ERG25 | K07750 | IZ007625 | DESC | K00507 |
| IZ002623 |  | K07735 | IZ003793 | DESC | K00507 |
| IZ002428 | MUTS2 | K07456 | IZ003630 | CYP51 | K00493,K05917,K14338,K15470 |
| IZ011857 | FHAB | K07326,K15125 | IZ010626 |  | K00472 |
| IZ007228 | MSRB | K07305 | IZ002335 |  | K00432 |
| IZ010091 |  | K07305 | IZ001893 | YUXK |  |
| IZ014884 |  | K06995 | IZ011516 | PGUG_04313 | |
| IZ005638 | FALCILYSIN | K06972 | IZ007836 | OSI_16281 | |
| IZ014477 | LGALS3 | K06831 | IZ004084 | NUGGC |  |
| IZ013937 | TYPA | K06207 | IZ001229 | NLRC3 |  |
| IZ014596 | LYPLA2 | K06130 | IZ003687 | NLRC3 |  |
| IZ012706 | YDFG | K05886,K15373,K16066 | IZ012713 | CYB5D2 |  |
| IZ001959 |  | K04773 | IZ004136 | CBWD1 |  |
| IZ010162 | RBL1 | K04681,K06618,K16332 | IZ006127 | C2ORF81 |  |
| IZ000514 |  | K04456,K04688,K08282,K13302,K13303 | IZ012689 | C22ORF23 | |

Table S14 Annotation for up- regulated DEGs in A-W5d vs. A-G5d.

| geneID | Name | KO number | geneID | Name | KO number |
| --- | --- | --- | --- | --- | --- |
| IZ007510 | ZNHIT6 |  | IZ009977 | NUDT17 |  |
| IZ007954 | ZDHHC15 | K14423,K18932 | IZ006051 | NUDCD3 |  |
| IZ014828 | ZDHHC14 | K07542,K16675 | IZ003688 | NUC | K01174 |
| IZ013677 | YXJI |  | IZ001920 | NSUN2 | K15335 |
| IZ002107 | YKRP |  | IZ008375 | NRDA | K00525 |
| IZ001626 | YHDJ | K00571,K07319 | IZ003687 | NLRC3 |  |
| IZ001693 | YFEW |  | IZ013821 | NLRC3 |  |
| IZ004959 | YEAD | K01792 | IZ001229 | NLRC3 |  |
| IZ012706 | YDFG | K05886,K15373 | IZ005716 | NLRC3 |  |
| IZ010809 | YBEQ | K07126 | IZ011907 | NDH | K03885 |
| IZ007381 | XPNPEP3 | K01262 | IZ013960 | MYO2 | K10357 |
| IZ009095 | WTAP |  | IZ014812 | MYO10 | K12559 |
| IZ014894 | WPRA | K13274,K14645 | IZ009993 | MT0155 | K00128 |
| IZ000459 | WDR81 | K17601 | IZ002431 | MLYCD | K01578 |
| IZ003390 | WDR53 |  | IZ000084 | MKS1 | K03030,K19332 |
| IZ014085 | WBNF | K01784,K08679 | IZ005971 | MGL1 | K01739,K01761 |
| IZ011498 | VPS52 |  | IZ004734 | METF | K00297 |
| IZ003477 | VPS51 |  | IZ000453 | MCM7 | K02210 |
| IZ013337 | UXUB | K00040,K00045 | IZ014752 | MCM6 | K02542 |
| IZ012846 | UVR8 |  | IZ013928 | MANF | K13190 |
| IZ001180 | URI1 | K17560 | IZ002113 | LRRC56 |  |
| IZ008730 | UGP1 | K00963,K01835 | IZ003248 | LRRC49 | K16606 |
| IZ004225 | UGD2 | K00012 | IZ011428 | LMLN | K01404,K08654 |
| IZ003978 | UBR2 | K10625,K10626 | IZ007080 | LMAN2 | K10082,K10083 |
| IZ001687 | UBIAD1 |  | IZ012845 | LIP1 | K03644 |
| IZ004482 | UBE3C | K10589 | IZ014314 | LID | K11446 |
| IZ013937 | TYPA | K06207 | IZ003673 | LHCX2 | K08907 |
| IZ009236 | TXNL4A | K12859 | IZ003671 | LHCX2 | K08907 |
| IZ013576 | TXNDC5 | K13984 | IZ001085 | LHCX2 | K08907 |
| IZ001672 | TTPAL |  | IZ004582 | LHCB5 | K08916 |
| IZ011799 | TTN | K12567 | IZ014477 | LGALS3 | K06831 |
| IZ002322 | TSSC1 |  | IZ004663 | LEPA | K03596 |
| IZ005120 | TSR1 | K14799 | IZ009793 | KLHL2 | K10443 |
| IZ011379 | TSF | K02357 | IZ011381 | KIF27 | K10395,K18806 |
| IZ000789 | TRPM2 | K04977,K04979 | IZ000556 | KIAA1919 | K08175 |
| IZ013088 | TRMT11 | K15430 | IZ002250 | KCO1 | K05389 |
| IZ006674 | TRM1 | K00555 | IZ008164 | ITPKA | K00911 |
| IZ006591 | TRAF3IP1 | K19680 | IZ012005 | INPPL1 |  |
| IZ002881 | TOLA | K03646 | IZ012386 | INPP5D | K03084,K15909 |
| IZ014362 | TOLA | K03646 | IZ003339 | INFB | K02519 |
| IZ010408 | TOLA | K03646 | IZ012893 | HSPF | K13993 |
| IZ011253 | TOLA | K03646 | IZ012892 | HSPF | K13993 |
| IZ008942 | TOLA | K03646 | IZ009267 | HSP90B | K09487 |
| IZ005545 | TOLA | K03646 | IZ000620 | HSP70 | K03283 |
| IZ010669 | TMS1 |  | IZ006133 | HSP104 | K03695 |
| IZ001836 | TMEM144 | | IZ009655 | HSF2 | K09414,K09415 |
| IZ011051 | TMBIM6 |  | IZ013154 | HOP | K09553 |
| IZ005290 | THUMPD2 | | IZ000102 | HM13 | K09595 |
| IZ012114 | TETA | K08151 | IZ004789 | HID1 |  |
| IZ008489 | TCEA1 | K03145,K04533,K15169 | IZ011423 | HERC2 | K10595 |
| IZ003595 | TBF1 |  | IZ005987 | HERC2 | K10595 |
| IZ003298 | TAL | K00616 | IZ008378 | HECTD2 | K10587,K10614 |
| IZ012338 | SYVN1 | K10601,K17262 | IZ007817 | HBS1L | K14416 |
| IZ011235 | SYNJ1 | K01099 | IZ003577 | GYLTL1B | K09668 |
| IZ009381 | SURF4 |  | IZ013830 | GSTCD |  |
| IZ012370 | SUPT4H1 | K15171 | IZ009191 | GPMB | K01834,K15634 |
| IZ013839 | STX5 | K08490 | IZ000647 | GPATCH1 | K13123 |
| IZ004766 | STK33 | K08813 | IZ000552 | GLPK | K00864 |
| IZ005043 | ST5 |  | IZ010777 | GLNS | K01886 |
| IZ013493 | SSEA | K01011,K07112 | IZ002472 | G9A | K11420 |
| IZ004420 | SRD5A1 | K12343 | IZ002202 | FUCU | K02431 |
| IZ014607 | SPRYD7 |  | IZ006532 | FTSY | K03110 |
| IZ009372 | SPOVK |  | IZ014045 | FTSY | K03110 |
| IZ009065 | SPNS3 | K09008,K12890 | IZ011578 | FTSH | K03798 |
| IZ003845 | SPG1 | K06682,K07976 | IZ008412 | FOLC | K11754 |
| IZ011572 | SMC4 | K06675 | IZ011414 | FKS1 | K00706 |
| IZ012674 | SLC35A2 | K15272 | IZ001633 | FKPA | K01802,K03772 |
| IZ003971 | SLC35A2 | K15272 | IZ006959 | FIBP |  |
| IZ009165 | SLC25A1 | K15100 | IZ005109 | FGSG_07719 | |
| IZ002008 | SLC13A5 | K14445 | IZ004868 | FG11507.1 | |
| IZ000886 | SETD6 | K05302 | IZ009366 | FG10381.1 | K06683 |
| IZ010920 | SEL1L | K14026 | IZ009392 | FG09158.1 | K17800 |
| IZ012656 | SEC14L4 |  | IZ010529 | FG08746.1 | |
| IZ010678 | SCP2D1 | K12405 | IZ011436 | FG07931.1 | K05768 |
| IZ001685 | SCO1 | K07152 | IZ014514 | FG07607.1 | |
| IZ009130 | SCLAV_5304 | | IZ002541 | FG05589.1 | |
| IZ001340 | SCLAV_2631 | K00729 | IZ004110 | FG05244.1 | K14763 |
| IZ003003 | SCAT | K17600 | IZ014761 | FG02130.1 | |
| IZ008491 | SART1 | K11984 | IZ004023 | FG02130.1 | |
| IZ002906 | SAE2 | K10685 | IZ003832 | FG01886.1 | K15559 |
| IZ002511 | SAC1 |  | IZ014896 | FG01884.1 | K01054 |
| IZ005770 | RTF1 | K15178 | IZ004559 | FG01373.1 | |
| IZ014268 | RTDR1 |  | IZ007975 | FG00050.1 | |
| IZ013697 | RSMI | K07056 | IZ012461 | FANCI | K10895 |
| IZ012665 | RPT1A | K03061 | IZ010224 | FAM18A | K00948 |
| IZ000875 | RPSB | K02967 | IZ008851 | FAM18A |  |
| IZ005884 | RPLV | K02890 | IZ012797 | FAM151A | |
| IZ006221 | RPL38 | K02923 | IZ005638 | FALCILYSIN | K06972 |
| IZ007234 | RPGRIP1 | K16512,K16550 | IZ006909 | FADS1 | K10224,K10225,K10226 |
| IZ008230 | RPGR | K19607 | IZ000161 | ENTPD1 | K01510 |
| IZ000760 | RLUC | K06175,K06177 | IZ011479 | EIF2AK1 | K16194 |
| IZ011761 | RHDA | K01011,K07112 | IZ006848 | EIF2AK1 | K16194 |
| IZ010735 | RCE1 | K08658 | IZ013437 | EI24 | K10134 |
| IZ001286 | RBX1 | K03868 | IZ008469 | EHMT1 | K11420 |
| IZ003013 | RBSK | K00852 | IZ000679 | ECHDC1 | K18426 |
| IZ010502 | RBMX2 | K13107 | IZ012586 | ECA2 | K01537,K05853 |
| IZ013600 | RAB8 | K07901 | IZ009417 | ECA2 | K01537,K05853 |
| IZ004649 | RAB6 | K07893 | IZ013906 | DNMT2 | K15336 |
| IZ012384 | PUX3 | K14012 | IZ006568 | DNLZ | K17808 |
| IZ003507 | PUC | K04459 | IZ002027 | DNAJC22 | K19370 |
| IZ008835 | PTRB | K01322,K01354 | IZ011054 | DNAJC11 | K09531 |
| IZ003012 | PTK2 | K08286 | IZ011915 | DNAAF3 | K19752 |
| IZ003192 | PTCHD2 | K10649,K18730 | IZ003091 | DLD | K00102,K00104 |
| IZ000029 | PSMD5 | K06692 | IZ000783 | DHDDS | K11778 |
| IZ007930 | PSMD2 | K03028 | IZ002412 | DERL2 | K13989 |
| IZ000031 | PROP | K03762,K12226 | IZ007051 | DCM | K00571,K03497 |
| IZ007305 | PROB | K00931 | IZ008219 | D-CDES |  |
| IZ008053 | PPP4R1 | K15424 | IZ005448 | DAGLB | K13806 |
| IZ003819 | PPIF | K03767,K09565 | IZ009491 | CYP9 | K01802 |
| IZ000855 | PPA2 | K01507,K06094 | IZ005432 | CYP51 | K00493,K05917 |
| IZ004632 | POLI | K03510 | IZ011740 | CYFIP2 | K05749 |
| IZ005994 | PNCB |  | IZ012713 | CYB5D2 |  |
| IZ006173 | PLEC | K10388,K19827 | IZ009213 | CTPA | K03797 |
| IZ006592 | PLCH2 |  | IZ009534 | CSRP2 | K09377 |
| IZ000691 | PLCE1 | K05860 | IZ011724 | CPN60A | K04077 |
| IZ011083 | PKHD1L1 | K19865 | IZ014496 | CPN20 | K04078,K09494 |
| IZ003291 | PIP | K01259 | IZ005218 | CPK20 | K13412 |
| IZ008880 | PIM1 | K08675,K11086 | IZ007114 | CPI1 | K08246 |
| IZ010119 | PHZC |  | IZ001710 | CP3 | K01365,K01371 |
| IZ000376 | PHOT2 | K08282 | IZ003831 | CLPB | K03695,K03696 |
| IZ008413 | PGUG_05820 | K00471 | IZ001352 | CLIP1 | K10421 |
| IZ004071 | PGUG_05407 | | IZ001387 | CLEC16A | K19513 |
| IZ011516 | PGUG_04313 | | IZ014208 | CHM | K11450,K17255 |
| IZ009751 | PGUG_04221 | K03809,K14015 | IZ012216 | CEP76 | K16457 |
| IZ014524 | PGUG_03509 | K05012 | IZ011233 | CDC37 | K09554 |
| IZ004885 | PGUG_02756 | | IZ011938 | CDC20.1 | K03363,K15028 |
| IZ002763 | PGUG_02249 | K00147 | IZ005262 | CCDC151 |  |
| IZ013178 | PGGT1B | K11713 | IZ011183 | CCA1 | K00974 |
| IZ003980 | PGAM5 | K15637 | IZ000467 | CC2D2A | K19352 |
| IZ004337 | PDS5B | K11267 | IZ004136 | CBWD1 |  |
| IZ004521 | PDIA4 | K09582 | IZ005902 | CALR | K08057 |
| IZ012779 | PDIA3 | K08056 | IZ006127 | C2ORF81 |  |
| IZ008977 | PDI1 | K09580 | IZ012689 | C22ORF23 | |
| IZ004968 | PDCD5 | K06875 | IZ006266 | BRD3 | K11721 |
| IZ004407 | PCTP |  | IZ004628 | BMUL_2604 | |
| IZ012044 | PAQR4 |  | IZ000319 | BMUL_2369 | K12600 |
| IZ001378 | OTC | K00611 | IZ013774 | BMUL_2369 | K12600 |
| IZ012626 | OSI_37591 | | IZ011409 | BMUL_2369 | K09134,K12600 |
| IZ014672 | OSI_37533 | | IZ004710 | BIP | K09490 |
| IZ000417 | OSI_35102 | | IZ002737 | B9D2 | K16745 |
| IZ005990 | OSI_26669 | | IZ014189 | ATG9 | K17907 |
| IZ007981 | OSI_21267 | K05941 | IZ008126 | ATG5 | K08339 |
| IZ002543 | OSI_21267 | K05941 | IZ004601 | ATF7IP |  |
| IZ012458 | OSI_18234 | K11271 | IZ013384 | ASPG | K01424,K13278 |
| IZ004862 | OSI_16973 | | IZ009273 | ASNA | K01914 |
| IZ002275 | OSI_09353 | | IZ008040 | ARL1 | K07942 |
| IZ012328 | OSI_07437 | | IZ013670 | ANP32B | K18647 |
| IZ000784 | OSI_06346 | K12823 | IZ002955 | ANO3 | K19498,K19499 |
| IZ012648 | OSI_06299 | K01802,K09566 | IZ011146 | AMMECR1 | |
| IZ000892 | OSI_03766 | | IZ001452 | ALG11 | K03844 |
| IZ007782 | ORC5 | K02607 | IZ010454 | AKR5F | K06221 |
| IZ000900 | OCAR_7038 | K00641 | IZ013809 | ACR-25 |  |
| IZ011957 | OCAR_4293 | K03286 | IZ004634 | ACAA1 | K07513,K10408 |
| IZ011486 | NUSG | K02601 | IZ000987 | ABHD6 | K13700 |
